# The Treatment of Obesity in the Context of the Obesity Paradox in Patients with Heart Failure: A Narrative Review

**DOI:** 10.1007/s11886-025-02333-5

**Published:** 2025-12-16

**Authors:** Pamela L. Alebna, Sarah Martey, Anurag Mehta, Carl J. Lavie, Salvatore Carbone

**Affiliations:** 1 Pauley Heart Center, Virginia Commonwealth University, Richmond, VA USA; 2 School of Medicine, Virginia Commonwealth University, Richmond, VA USA; 3 Division of Cardiology, Department of Medicine, Emory University School of Medicine, Atlanta, GA USA; 4 Ochsner Clinical School, John Ochsner Heart and Vascular Institute, The University of Queensland School of Medicine, New Orleans, LA USA; 5 Nutrition Program, EVMS School of Health Professions, Macon & Joan Brock Virginia Health Sciences at Old Dominion University, Norfolk, VA USA; 6 Division of Endocrine and Metabolic Disorders, Strelitz Diabetes Center, Department of Medicine, Eastern Virginia Medical School, Macon & Joan Brock Virginia Health Sciences at Old Dominion University, 855 W. Brambleton Ave, Norfolk, VA USA; 7 Nutrition Program, EVMS School of Health Professions, Division of Endocrine and Metabolic Disorders, Strelitz Diabetes Center, Department of Medicine, Eastern Virginia Medical School, Macon & Joan Brock Virginia Health Sciences at Old Dominion University, 855 W. Brambleton Ave, Room 229, Norfolk, VA 23510-1001 USA

**Keywords:** Anti-obesity medications, Heart failure, HFpEF, Cardiorespiratory fitness, Obesity paradox, Obesity

## Abstract

**Purpose of review:**

While excess adiposity is a known risk factor for incident heart failure ( HF), once the condition is established, observational data suggest that increased body mass index (BMI) may confer a survival advantage. This paradox has emphasized the underlying roles of cardiorespiratory fitness (CRF), and body composition, particularly lean mass (LM), in influencing clinical outcomes.

**Recent findings:**

In this review, we explore the multifaceted nature of the obesity paradox in HF, with a focus on emerging anti-obesity incretin-mimetic therapies, such as glucagon-like peptide-1 receptor agonists (GLP-1 RAs) and dual GLP-1/glucose-dependent insulinotropic polypeptide (GIP) receptor agonists. These agents have demonstrated remarkable efficacy in weight reduction and favorable cardiovascular profiles in patients with HF with preserved ejection fraction (EF), yet their use in other HF populations, such as HF with reduced EF, raises important clinical questions and the urgent need for future research. Concerns include the potential for LM loss, implications for sarcopenic obesity, and the uncertain impact of weight loss on outcomes in patients who may not benefit from weight loss. We also highlight the need to assess therapeutic outcomes beyond BMI, incorporating measures of CRF, such as peak oxygen consumption (VO₂ peak), quality of life, and functional capacity, using tools such as the 6-minute walk test. Barriers to implementation, including cost, provider hesitation, insurance restrictions, and patient level challenges are also reviewed.

**Summary:**

Finally, we call for future research using contemporary cohorts and advanced phenotyping to reevaluate the obesity paradox in the context of modern pharmacologic interventions. As obesity treatment continues to evolve, a patient-centered, individualized approach that integrates body composition, functional status, and comorbid conditions will be essential in optimizing care for individuals with HF.

## Introduction

Obesity is one of the most significant health threats of the 21 st century. Once primarily confined to high-income countries, it has now become a global issue, increasing inprevalence in low- and middle-income countries [1]. In these settings, obesity adds to the existing burden of communicable diseases, creating a double burden of disease [2]. Since the 1980 s, the incidence of obesity in the United States has shown a persistent upward trajectory [3]. In wealthier nations, it exacerbates healthcare disparities, disproportionately impacting socioeconomically disadvantaged populations [4]. This rising prevalence is largely driven by a global shift toward sedentary lifestyles and increased consumption of energy-dense, nutrient-poor diets [5]. Beyond its health consequences, obesity imposes significant social, behavioral, and financial burdens. For example, in 2023, Medicaid expenditures on anti-obesity medications (AOM) reached historic highs at $3.9 billion [6]. Obesity in adolescence is also linked to poorer academic performance and long-term psychosocial challenges [7].

While body composition assessment is required to determine whether an excess adiposity is present, body mass index (BMI) remains the most widely used metric in clinical practice [8]. Despite its utility, BMI cannot distinguish fat from lean mass (LM) or inform fat distribution in the body, limitations that have important implications for disease risk stratification [9]. Emerging evidence emphasizes the importance of fat distribution, with metabolically active depots such as visceral adipose tissue (VAT) and epicardial fat playing key roles in cardiometabolic disease [10]. Importantly, individuals with a normal BMI may still exhibit excess visceral fat, a phenomenon known as “normal weight obesity” [11].VAT is strongly associated with systemic inflammation and the development of heart failure (HF), especially HF with preserved ejection fraction (EF; HFpEF) [12]. As the definition of obesity evolves, there is growing recognition of the need to differentiate between “preclinical obesity”, characterized by excess adiposity without overt organ dysfunction and “clinical obesity,” where obesity is associated with metabolic or functional impairment [13]. This distinction has important implications for treatment selection, monitoring, and therapeutic goals.

Obesity is a well-established risk factor for multiple cardiovascular (CV) conditions, including hypertension, type 2 diabetes, stroke, venous thromboembolism, and HF [14, 15]. Paradoxically, while obesity increases the risk of developing HF, several observational studies have shown that among individuals with established disease, those who are overweight or have class I-II obesity experience more favorable survival compared to their normal or underweight counterparts [16, 17], despite a greater risk for HF-related hospitalizations. This counterintuitive phenomenon, known as the obesity paradox, has been consistently reported across multiple HF populations [18, 19]. Proposed explanations are discussed in the following sections.

## The Obesity Paradox in HF

The obesity paradox was first described in the early 2000 s [16], and suggests that individuals with HF who are overweight or have class I-II obesity tend to have better short-term survival outcomes compared to their normal and underweight counterparts. This phenomenon has been documented in multiple observational studies [18–20]. For example, in a meta-analysis involving 22,807 HF with reduced EF (HFrEF) patients and a mean follow-up of 2.85 years, the risk of CV disease (CVD) mortality and HF hospitalizations (HFH) was lowest among individuals in the overweight category, relative risks (RR) of 0.79 (95% CI: 0.70–0.90) and 0.92 (95% CI: 0.86–0.97), respectively. In contrast, underweight individuals had the highest risk of adverse outcomes, with CVD mortality and HFH RRs of 1.20 (95% CI: 1.01–1.43) and 1.19 (95% CI: 1.09–1.30), respectively [21].

Despite the evidence supporting the obesity paradox [18, 19], it remains a topic of debate [22]. Critics argue that the apparent survival benefit may be confounded by factors, such as better metabolic reserves in overweight individuals, rather than a true protective effect of excess adiposity. Moreover, these patients may be more tolerant of aggressive treatment or have different disease phenotypes [23–25].

Just like in HFrEF, the obesity paradox in HFpEF has been observed [26, 27]. HFpEF is a clinical syndrome characterized by signs and symptoms of HF despite a preserved left ventricular (LV) EF (LVEF ≥ 50%) [28]. It is often associated with elevated LV filling pressures, impaired diastolic function, and exercise intolerance [29]. Obesity is a particularly prominent risk factor for HFpEF, data suggest that up to about 80% of individuals with HFpEF have obesity [30, 31]. In a meta-analysis of over 15,000 cases of HF, a 5-unit increase in BMI was associated with a HR of 1.41 (95% CI, 1.34–1.47) for developing HF [32], although no single BMI threshold has been universally accepted.

The pathophysiological link between obesity and HFpEF is complex and multifactorial. Excess adiposity activates the sympathetic nervous system and renin-angiotensin-aldosterone system (RAAS) and promotes the release of pro-inflammatory adipokines. These changes contribute to chronic systemic inflammation, oxidative stress, and myocardial remodeling. Obesity also induces structural cardiac changes, including LV hypertrophy, interstitial fibrosis, and impaired myocardial relaxation [30, 33, 34]. These alterations result in elevated filling pressures and the characteristic hemodynamic derangements of HFpEF. Notably, the risk of developing HF appears to increase in a dose-dependent manner with increasing obesity categories.

### Role of Cardiorespiratory Fitness (CRF) in the Obesity Paradox

CRF refers to the body’s ability to deliver and utilize oxygen efficiently during sustained physical activity. It is typically assessed using cardiopulmonary exercise testing (CPX) and expressed in metabolic equivalents (METs) or peak oxygen consumption (VO_2 peak_) [35]. However, due to limited availability of CPX in routine clinical practice, surrogate assessments, such as the exercise treadmill test (which gives estimated METs) or the six-minute walk test, are commonly used. CRF is a robust marker of CV health, with higher levels associated with improved diastolic function [36], lower blood pressure [37], reduced peripheral resistance, and ultimately, decreased morbidity and mortality [38].

In patients with HF and obesity, CRF plays a critical role in modifying outcomes. Individuals with high CRF have better survival compared to their counterparts with low CRF, independent of their BMI. In a study by Lavie et al. involving 2,066 patients with systolic HF, the obesity paradox was not evident among those with high CRF. However, among those with low CRF, BMI remained a significant predictor of mortality over a five-year follow-up period, even after adjusting for age and sex [39]. Similarly, findings from the Veterans Exercise Testing Study (VETS) revealed that, after adjusting for METs achieved, the survival advantage previously observed in individuals with obesity was no longer significant [40]. These findings suggest that CRF may help explain the obesity paradox, wherein overweight or class I-II obesity individuals exhibit better outcomes than those of normal weight.

## Weight Loss Therapies in HF

The role of weight loss in HF is an area of active research [41]. Intentional weight loss improves symptoms, functional status, and hemodynamics in patients with obesity and HFpEF. Weight loss, particularly through surgical interventions such as bariatric surgery, has been associated with a lower incidence of HF in the general population [42]. In a propensity matched retrospective study of Medicare beneficiaries with HF and obesity, bariatric surgery and pharmacotherapies were associated with lower risk of all-cause mortality and HFH with HR’s of 0.55 (95% CI, 0.49–0.63) and 0.72 (95% CI, 0.67–0.77), respectively; notably the study included only patients with Class II obesity and above [43]. However, it is unclear what extent of intentional weight loss confers a survival advantage once HF is established, especially among those with HFrEF, who are overweight or have class I obesity.

Over the past decade, significant advancements have been made in the development of anti-obesity therapies, particularly with the emergence of the incretin-mimetic therapies, such as glucagon-like peptide-1 receptor agonists (GLP-1 RAs). The approval of liraglutide following the SCALE trial in 2014 marked a turning point [44], followed by semaglutide in the STEP trials [45] beginning in 2021, and more recently, tirzepatide through the SURMOUNT trials in 2022. These agents have demonstrated impressive weight loss efficacy, achieving average reductions in total body weight of approximately 8%, 16%, and 20% compared to baseline, respectively [46]. 

Beyond their weight-lowering capabilities, these medications appear to confer CV benefits through mechanisms that extend beyond weight reduction alone. In the SELECT trial [47], which evaluated semaglutide in individuals without diabetes, the curves for CVD outcomes, such as HFH and health status measured by the Kansas City Cardiomyopathy Questionnaire (KCCQ) began to separate early, prior to the onset of substantial weight differences between treatment groups. This observation suggests that GLP-1 RAs may exert beneficial effects through pathways, such as improved endothelial function, microvascular remodeling, and anti-inflammatory activity [48, 49]. Emerging evidence also suggests improvements in comorbid conditions, including obstructive sleep apnea [50]. 

In the context of HFpEF, weight loss therapies hold promise. The STEP-HFpEF trial, which enrolled patients with HFpEF and a BMI greater than 30 kg/m², demonstrated that once-weekly semaglutide not only led to significant weight reduction but also improved symptoms, physical function, and quality of life (QoL). This was reflected in a significant improvement in the KCCQ score, with a mean difference of 7.8 points (95% CI, 4.8 to 10.9; *P* < 0.001) compared to placebo [51]. Similarly, the SUMMIT trial, which included 364 patients with HFpEF and obesity, found that treatment with tirzepatide resulted in a 38% reduction in the incidence of the composite outcome of CVD death and HFH events compared to placebo [52]. The STEP-HFpEF-DM trial, which focused on patients with HFpEF, obesity (BMI >30 kg/m²), and T2D, also reported favorable outcomes with semaglutide. Participants in the semaglutide arm experienced greater weight loss and improved patient-reported outcomes, with a mean KCCQ score increase of 7.3 points (95% CI, 4.1 to 10.4; *P* < 0.001). Some of these improvements might be independent of weight loss and thought to be mediated by favorable changes in hemodynamics, LV stiffness, and systemic inflammation. However, the growing use of these therapies raises important questions regarding the obesity paradox observed in HF. As potent weight loss agents, such as GLP-1 RAs, become more widely used in this population, it remains unclear how intentional weight reduction might influence outcomes in patients with heart failure particularly those with HFrEF, where the obesity paradox appears most pronounced. Concerns have also been raised about potential adverse effects, such as the disproportionate loss of lean mass, which could impair functional capacity and possibly worsen frailty in vulnerable individuals [53]. These considerations underscore the need for an individualized approach when prescribing weight loss therapies in patients with HF. Future studies are needed to determine the optimal degree of weight loss, the role of body composition monitoring, and the long-term impact of these medications on morbidity and mortality in this population.

## Weight Loss with AOM in HF: Focus on Body Composition

In evaluating the potential benefits of overweight and class I-II obesity in HF, increasing attention has been paid to the role of body composition particularly CRF and skeletal muscle mass (SMM) in mediating improved outcomes. Body composition, rather than weight alone, may be a better determinant of prognosis and QoL in patients with HF [19, 54–56]. In individuals with excess adiposity, greater SMM has been independently associated with improved survival and better exercise capacity [57]. SMM contributes significantly to CRF, metabolic reserve, and physical resilience. Conversely, the presence of both reduced SMM and excess fat known as sarcopenic obesity (SO) has been linked to worse functional status, increased frailty, and poorer outcomes in HF patients [58]. 

**Fig. 1:**
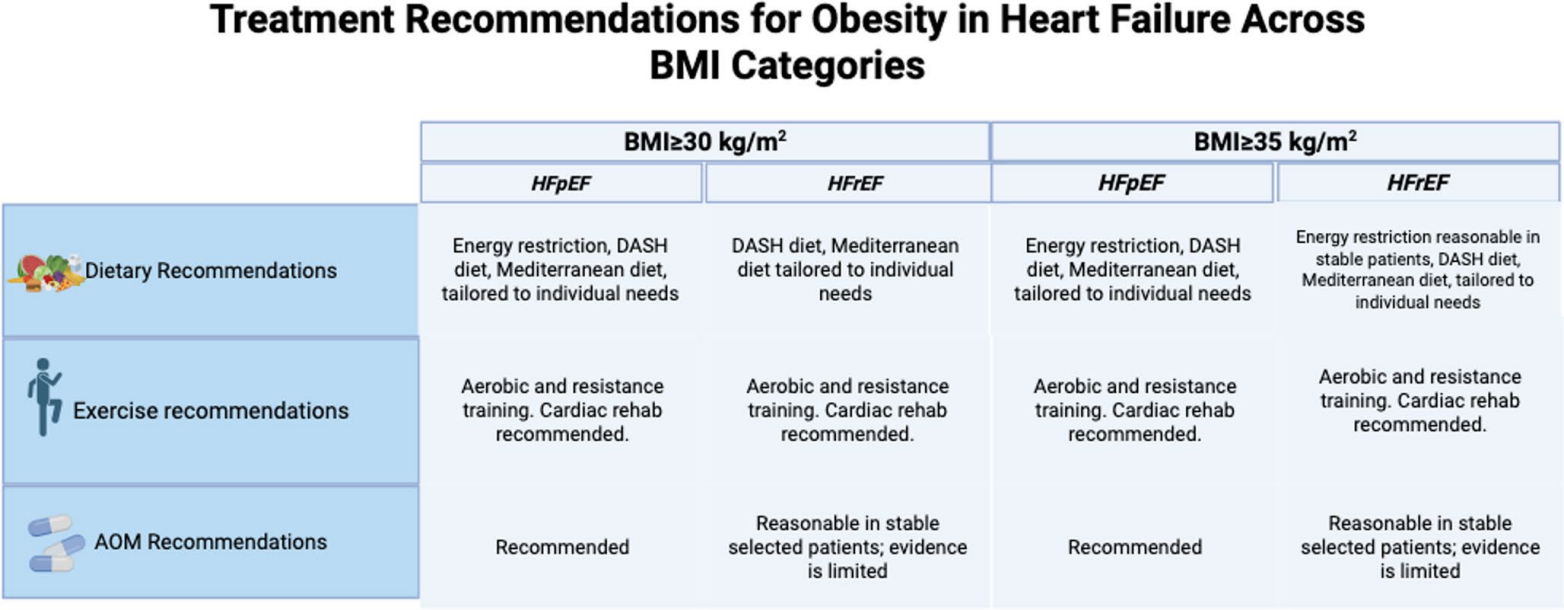
This figure summarizes dietary, exercise, and pharmacologic recommendations for the treatment of obesity in patients with heart failure across two BMI categories: ≥ 30 and ≥ 35 kg/m². Recommendations are stratified by heart failure subtype—heart failure with preserved ejection fraction (HFpEF) and heart failure with reduced ejection fraction (HFrEF) AOM- Anti-obesity medication; BMI- body mass index

Sarcopenia can result from multiple factors, including chronic inflammation, inadequate nutrition, comorbid conditions, and physical inactivity. Exercise, especially resistance training [55], remains a key intervention to preserve or improve SMM quantity and quality. However, the rise in pharmacologic weight loss strategies, particularly GLP-1 RAs and dual GLP-1/GIP receptor agonists such as tirzepatide, has prompted new concerns about the unintended loss of LM [59]. AOMs have shown remarkable efficacy in reducing total body weight, but these reductions do not distinguish between fat mass and LM. Studies suggest that significant portions of the weight lost with GLP-1 RAs and dual agonists may involve fat-free mass (FFM), which includes SMM. For example, in a comparative study evaluating body composition changes, tirzepatide and semaglutide were both associated with significant reductions in FFM. Tirzepatide resulted in a greater FFM loss compared to placebo (− 1.5 kg; 95% CI: −2.3 to − 0.7; *P* < 0.001) and to semaglutide (− 0.8 kg; 95% CI: −1.5 to − 0.1; *P* = 0.018) [60]. The implications of this LM loss are particularly concerning in HF, where muscle mass is closely tied to physical function and survival. While strategies such as increased protein intake and structured resistance training [61] are commonly recommended to preserve muscle mass during weight loss, direct evidence supporting their effectiveness specifically during AOM use is limited. Nevertheless, these recommendations can be extrapolated from existing data on sarcopenia and exercise physiology (Fig. 1).

**Fig. 2:**
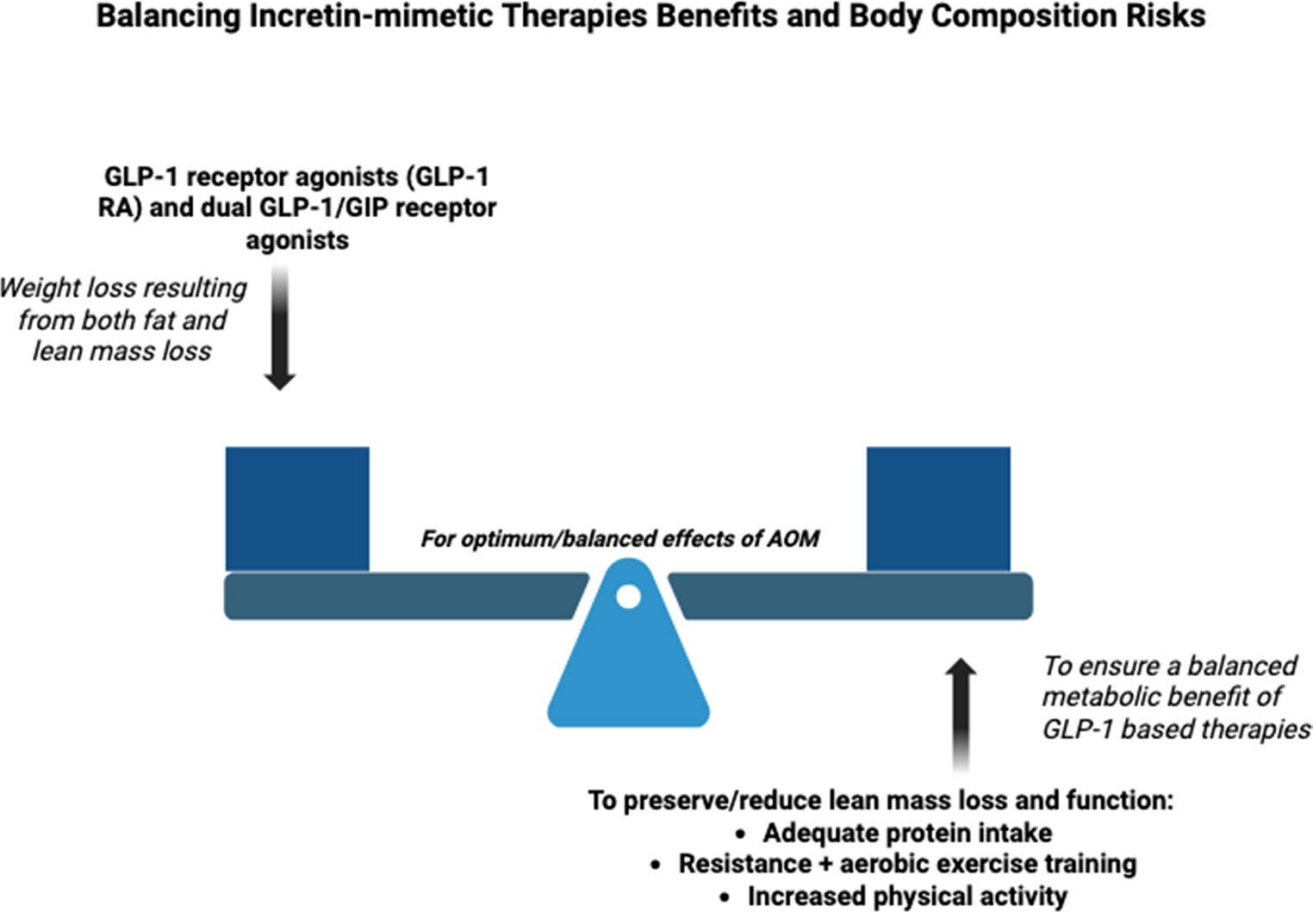
Figure 2 depicts the physiological balance between the metabolic benefits of GLP-1 and dual GLP-1/GIP receptor agonists (AOMs) and the potential unintended consequence of lean mass loss during weight reduction. Left side of the scale represents the weight-reducing effects of incretin-mimetic therapies. Right side of the scale illustrates the need for adequate dietary protein intake and structured resistance training which aim to preserve skeletal muscle mass or to prevent its loss, and physical function during use of medications for weight loss

Another important consideration is what happens if these agents are discontinued. Emerging data indicate a high likelihood of weight regain and potential metabolic rebound following cessation of therapy, which could lead to fluctuations in both fat and muscle mass [62]. These dynamics highlight the importance of long-term treatment planning, body composition monitoring, and individualized approaches to weight loss in patients with HF. While pharmacological weight loss agents offer significant promise, their effects on muscle mass and long-term metabolic health must be carefully considered particularly in vulnerable populations such as those with HF. Future studies should prioritize evaluating body composition changes and incorporating interventions to preserve lean mass or reduce its loss alongside weight reduction (Fig. 2).

## Assessing Outcomes with AOMs

While weight loss remains the primary endpoint in evaluating the efficacy of AOMs, a more comprehensive assessment must consider outcomes beyond BMI, especially when these agents are used to address obesity-related complications. When AOMs are prescribed for comorbid conditions for example T2D or obstructive sleep apnea, the focus is on changes in glycemic control or apnea-hypopnea index. However, in patients with obesity and chronic conditions such as HF, particularly older adults or those with sarcopenia, other meaningful outcomes should be prioritized. These should include CRF, physical function, and health-related QoL. Objective tools to assess these outcomes include the 6MWT, VO₂ _peak_, and validated patient-reported measures like the KCCQ. These metrics offer insights into functional capacity, symptom burden, and overall well-being that BMI alone cannot capture. This is especially relevant in patients at risk for LM loss or those with existing impairments in skeletal muscle function. As we move toward more personalized care, integrating multidimensional assessments into clinical trials and practice will help ensure that treatment benefits extend beyond weight loss to include functional and QoL improvements that matter most to patients.

## Barriers To Implementation of Weight Loss Therapies

Despite the efficacy of the newer AOMs, several barriers hinder their widespread adoption in clinical practice. These challenges can be broadly categorized into medication-related, provider-related, and system and patient level obstacles.

One of the most significant medication-related barriers is cost. The newer generation of AOMs such as GLP-1 receptor agonists (e.g., semaglutide) and dual GLP-1/GIP receptor agonists (e.g., tirzepatide) are expensive. As of 2024, the estimated cost to Medicare if all eligible beneficiaries were prescribed semaglutide was projected to reach $145 billion annually. The average out-of-pocket cost for tirzepatide is approximately $1,060 per month for uninsured individuals. Even for those with insurance, high co-pays and restrictive coverage policies often limit access. Prior authorization requirements and inconsistent insurance approval further complicate the prescribing process. Moreover, rising demand has led to medication shortages and a parallel market of compounded formulations, which may carry risks of dosing errors and impurities due to lack of standardization.

Provider-related barriers also contribute to underutilization. Many clinicians remain hesitant to prescribe AOMs, citing clinical inertia, unfamiliarity with evolving guidelines, and concern over long-term safety. There is also skepticism particularly in light of the obesity paradox observed in conditions like HF about the necessity of weight loss in all patients with obesity. Additionally, the need for ongoing monitoring, titration, and management of gastrointestinal side effects may deter providers from initiating therapy. These practical hurdles, combined with time constraints in routine practice, often limit discussions about obesity treatment.

At the policy level, restrictive insurance coverage and lack of recognition of obesity as a chronic disease by some payers result in inadequate reimbursement for both medications and multidisciplinary care. This undermines efforts to integrate obesity treatment into standard chronic disease management models. Patient-level barriers include low health literacy, lack of awareness about the effectiveness of modern therapies, fear of side effects, limited motivation, and the burden of managing multiple comorbidities. For many patients, stigma and past negative experiences with weight loss further reduce engagement with treatment. Addressing these multifaceted barriers will require a concerted effort involving patient education, clinician training, health policy reform, and expanded insurance coverage to support equitable access to effective obesity care.

### Future Directions

The obesity paradox remains a compelling yet controversial clinical observation, particularly in populations with HF and other catabolic chronic conditions. As clinical management evolves and more patients gain access to AOMs, such as GLP-1 receptor agonists and dual GLP-1/GIP receptor agonists, it is essential to re-evaluate the validity and clinical relevance of the paradox in contemporary settings. Existing evidence is largely derived from older cohorts and may not reflect the effects of modern therapies on body composition, CRF, or metabolic health. Future research should focus on prospective trials and real-world studies that assess not only weight loss, but also changes in LM, CRF, functional capacity, and QoL. These studies should include diverse patient populations, particularly those with HfpEF or HFrEF, to understand how intentional weight loss interacts with disease progression and outcomes. Moreover, integrating more sophisticated measures of body composition and functional status beyond BMI will help clarify the mechanisms underlying the paradox.

## Conclusions

In this review, we examined the obesity paradox and its implications for patients with HF, a population in which excess weight has paradoxically been associated with improved outcomes. We explored how factors such as cardiorespiratory fitness, skeletal muscle mass, and body composition may contribute to this phenomenon, suggesting that the quality and distribution of weight may be more relevant than weight alone. We also discussed the growing role of newer AOMs, particularly GLP-1 receptor agonists and dual GLP-1/GIP receptor agonists, which have demonstrated significant efficacy in weight reduction and potential CVD benefits. However, their use in HF raises critical questions about how intentional weight loss especially loss of LM might affect clinical outcomes in a population where the obesity paradox has been observed. As these therapies become increasingly integrated into clinical care, future research should focus on evaluating their long-term impact on mortality, function, and QoL in HF.

## Key References

- Rodrigues MM, Falcão LM. Pathophysiology of heart failure with preserved ejection fraction in overweight and obesity—Clinical and treatment implications. Int J Cardiol. 2025;430:133182. doi:10.1016/j.ijcard.2025.133182.
  - This review provides an overview of the mechanisms linking obesity to HFpEF, emphasizing the hemodynamic, inflammatory, and metabolic pathways involved and highlighting implications for tailored therapeutic strategies.
- Sanchis-Gomar F, Neeland IJ, Lavie CJ. Balancing weight and muscle loss in GLP1 receptor agonist therapy. Nat Rev Endocrinol. Published online July 28, 2025:1–2. doi:10.1038/s41574-025–01160-6.
  - This commentary highlights the importance of preserving lean mass during GLP-1 receptor agonist therapy, underscoring the need for concurrent lifestyle interventions to optimize cardiometabolic outcomes.

## Data Availability

No datasets were generated or analysed during the current study.

## Abbreviations

HFrEF: Heart Failure with Reduced Ejection Fraction
HFpEF: Heart Failure with Preserved Ejection Fraction
HF: Heart Failure
CRF: Cardiorespiratory Fitness
BMI: Body Mass Index
KCCQ: Kansas City Cardiomyopathy Questionnaire
AOM: Anti–Obesity Medications
